# Adjunctive vasopressors in distributive shock: How soon is early?

**DOI:** 10.1186/s13054-023-04500-y

**Published:** 2023-05-30

**Authors:** Bruno Garcia, Matthieu Legrand

**Affiliations:** 1 Department of Anesthesia & Peri-Operative Care, Division of Critical Care Medicine, University of California, San Francisco (UCSF), San Francisco, CA USA; 2 Department of Intensive Care, Centre Hospitalier Universitaire de Lille, Lille, France; 3 Experimental Laboratory of Intensive Care, Université Libre de Bruxelles, Brussels, Belgium

**Keywords:** Vasopressor, Septic shock, Vasopressin, Norepinephrine, Angiotensin II

## Background

Despite being commonly used in patients with distributive shock, the optimal strategy for vasopressor administration remains uncertain [1]. Recently Wieruszewski et al. reported in the journal an exploratory post-hoc analysis of the Angiotensin II for the Treatment of High-Output Shock (ATHOS-3) trial that initiation of angiotensin II at a low norepinephrine equivalent dose (NED) of ≤ 0.25 µg/kg/min was associated with higher likelihood of survival when compared to placebo [2]. The results of this study provide an opportunity to reflect on the available evidence regarding the link between the timing of second line vasopressors administration and outcomes in distributive shock.

## Adjuntive vasopressors: Timing or dose?

The administration strategy of vasopressors in distributive shock, particularly the impact of early and multimodal administration, is an area of interest due to its potential significant impact on outcome. When to initiate a second (or third) line vasopressor has long been debated. Most of the data available arises from the use of vasopressin [3, 4]. The addition of vasopressin to norepinephrine has not been demonstrated to improve outcomes in sepsis [5]. A meta-analysis of individual data from randomized trials found no association between vasopressin and improved survival or reduced organ failure rates [6] and the level of evidence supporting its use is considered insufficient for reimbursement in some countries [7].

Vasopressin is however recommended by the Surviving Sepsis Campaign for adults with septic shock who have inadequate mean arterial pressure (MAP) despite low to moderate doses of norepinephrine, but with a weak recommendation due to moderate quality evidence [4]. The suggestion to use vasopressin primarily stems from subgroup analyses of randomized trials and observational studies, which suggest better outcomes when vasopressin is initiated in less severe patients or those receiving lower doses of norepinephrine. In the VASST trial comparing the combination of norepinephrine and vasopressin to norepinephrine alone, patients who received less than 15 µg/min of NE showed better survival rates with the addition of vasopressin [5]. An additional observational study of 1610 patients with septic shock in the United States reported a 20.7% increase in in-hospital mortality for every 10 µg/min increase in norepinephrine-equivalent dose up to 60 µg/min at vasopressin initiation [8]. One proposed mechanism for the improved survival with lower-dose norepinephrine and vasopressin combination is a reduction in catecholamine exposure [8, 9]. The lower incidence of atrial fibrillation in randomized trials of vasopressin supports this hypothesis [6]. However, the potential role of vasopressin on improved outcomes may be more complex.

**Fig. 1:**
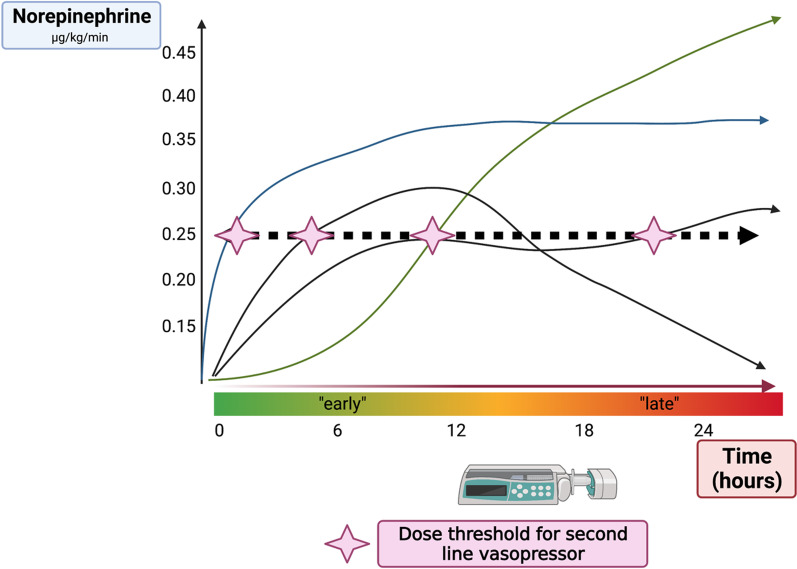
The examples show different trajectories of norepinephrine equivalent dose during vasodilatory shock. NED: Norepinephrine-equivalent dose. Variations in norepinephrine equivalent dose trajectories during vasodilatory shock can lead to differences in the timing of adjunctive vasopressor therapy. Depending on the case, treatment may be initiated soon after diagnosis or when hemodynamic stability improves, leading to difficulties to interpret the data

In the VAAST trial, the median interval between meeting the inclusion criteria and initiating vasopressin infusion was 11.9 h [5]. Similarly, in an observational retrospective study, the median time from shock onset to vasopressin administration was 5.3 h [8]. Altogether, most patients did receive norepinephrine for an extended period of time before vasopressin was started, challenging the “early” administration. A similar observation can be made regarding Angiotensin II: in the ATHOS-3 trial, one of the inclusion criteria was a minimum duration of norepinephrine use for at least 6 h [10]. Altogether, introducing a second line vasopressor (i.e. vasopressin) at a fixed threshold is not synonymous of early administration as the predefined threshold can be met at different times after onset of shock (Fig. 1). If a drug-specific protective effect exists, early administration may have a greater chance of improving outcomes compared to late administration. The evidence available from randomized trials does not provide sufficient data to conclude on this specific point.

## Norepinephrine equivalent dosing

Another level of complexity arises in the ATHOS-3 trial, as the majority of patients were already receiving norepinephrine and vasopressin at the time of randomization. This is particularly relevant as the cut-off between “low” and “high” vasopressors requirements included a calculation of NED.

The NED calculation implies a fixed conversion of vasopressin to NED (i.e. 0.04 U/min to 0.1 µg/kg/min), a mostly empiric converting table which may not adequately account for variations in vasopressin responsiveness. In ATHOS-3, 62.5% of the low-NED placebo group and 73.6% of the high-NED placebo group received vasopressin in the 6 h prior to randomization [10]. This is a critical aspect as higher doses of vasopressors (unsurprisingly) have previously been shown to correlate with worse that reflect higher severity of cardiovascular failure [11].

The observation that both the low-NED and high-NED placebo groups had similar mortality rates (48% vs 45%) raises questions about the ability of NED to fully capture the severity of cardiovascular failure in this study [2].

## Conclusions

The current evidence suggest that the addition of a second-line non-adrenergic vasopressor may lead to improved outcomes mostly in less severe septic patients receiving lower doses of vasopressors. While post-hoc analysis of available randomized trials provides valuable data for generating hypotheses, the design of available randomized trial does not allow to separate the best indication based on the timing (i.e., early) of introduction of vasopressors or the dose threshold of vasopressors. Additionally, the calculation of norepinephrine equivalent dose may not fully capture the severity of shock among patients receiving vasopressin and introduces a confounding factor in the interpretation of threshold for angiotensin II initiation in the ATHOS-3 trial.

Altogether, adaptative trials embedded into clinical workflow should be encouraged to provide an unbiased approach and inform on the best strategies for vasopressor use in patients with sepsis [12].

## Data Availability

Not applicable.

## Abbreviations

ATHOS 3: Angiotensin II for the treatment of high-output shock
MAP: Mean arterial pressure
NED: Norepinephrine-equivalent dose
SSC: Surviving sepsis campaign
VASST: Vasopressin and septic shock trial
